# E6 hijacks KDM5C/lnc_000231/miR‐497‐5p/CCNE1 axis to promote cervical cancer progression

**DOI:** 10.1111/jcmm.15746

**Published:** 2020-08-20

**Authors:** Yan Zhang, Xing Li, Jun Zhang, Lin Mao

**Affiliations:** ^1^ Department of Obstetrics and Gynecology Renmin Hospital of Wuhan University Wuhan China

**Keywords:** CCNE1, cervical cancer, KDM5C, lnc_000231, miR‐497‐5p

## Abstract

Emerging evidence suggests that long non‐coding RNA (lncRNA) plays an important role in disease development, particularly in cancers. Recent studies with genome‐wide sequencing on cervical squamous cell carcinoma and matched adjacent non‐tumour tissues showed that a newly identified lncRNA‐lnc_000231 was highly expressed in cervical cancers. However, the underlying mechanism through which it is activated and its role in cervical cancer progression is still unclear. In this study, first, we confirmed that lnc_000231 is up‐regulated in cervical cancer cells and tumour tissues. Mechanically, we demonstrated that E6 up‐regulates lnc_000231 expression through promoting its promoter region H3K4me3 modification by destabilizing KDM5C. In vitro and in vivo results showed that lnc_000231 promotes cervical cancer cell proliferation and tumour formation by acting as miR‐497‐5p sponge and maintaining cyclin E1 (CCNE1) expression. Thus, our studies identified a new signalling pathway through which E6 promotes cervical cancer progression. E6 hijacked KDM5C/lnc_000231/miR‐497‐5p/CCNE1 signalling pathway is a promising target for cervical cancer treatment in the future.

## 1 INTRODUCTION

The prevalence of cervical cancer poses great threat to women's health.^1^ Annually, over 500 000 patients were diagnosed with cervical cancer and approximately 300 000 died from this disease.^2^, ^3^, ^4^ Due to the widely use of papanicolaou test for early cervical cancer screening and the advancement of therapeutic methods such as surgery operation, chemical treatment and radiotherapy, the incidence of cervical cancer has been reduced by 40%‐50% in recent years.^5^, ^6^, ^7^ However, for those advanced cervical cancer patients, the 5‐year survival rate at the late stages can be as low as 15%.^8^, ^9^ Therefore, understanding the molecular mechanisms that initiate cervical cancer development and identifying new diagnostic markers are urgent for cervical cancer treatment, especially for those advanced cervical cancer patients.

Nearly, all cervical cancer cases are linked to human papillomavirus (HPV) infection. Human papillomavirus is a small DNA tumour virus, consisting of a circular DNA molecule enclosed with capsid proteins.^10^ Among all the genes encoded by HPV, E6 and E7 act as key oncogenes that promote tumour growth and malignant transformation.^11^, ^12^, ^13^, ^14^ Constitutive expression of E6/E7 immortalizes primary epithelial cells and promotes tumour formation in vivo.^15^ E7 interacts with and stabilizes retinoblastoma tumour suppressor family (RB1 and RB2), facilitating cell cycle transition from G1 to S phase.^16^ Similar to cell growth dysregulation, E6 interacting with ubiquitin ligase E6AP promotes tumour suppressor P53 degradation and cell proliferation.^17^ Despite HPV infection, other cofactors also contribute to cervical cancer progression, such as immunosuppression, smoking, co‐infection with HIV, co‐infection with *Chlamydia trachomatis* and herpes simplex virus type 2 and other probable cofactors.^12^

In addition to E6 and E7, other factors, such as non‐coding RNAs including long non‐coding RNA (lncRNA), microRNA (miRNA) and circular RNA, have been reported to play critical roles in cervical cancer development.^18^, ^19^ Long non‐coding RNA is defined as a group of RNAs ranging from about 200 bp to more than 100 kb in length with no or limited protein coding ability.^20^ Due to its abundance and specifically expressed in different cells, lncRNA has been linked to numerous cellular process such as cell proliferation, epigenetic regulation, long‐range DNA‐DNA interaction and miRNA sponge.^21^, ^22^, ^23^ Long non‐coding RNA regulates gene expression in a temporary and cell type‐specific manner and has been reported to serve as important targets for cancer diagnostics and therapeutics.^24^, ^25^

The rapid advancement of next‐generation sequencing provides an unprecedented opportunity for studying the roles of lncRNA in cancers. Recent studies with genome‐wide sequencing on cervical squamous cell carcinoma and matched adjacent non‐tumour tissues showed that, a newly identified lncRNA‐lnc_000231 was highly expressed in cervical cancers.^26^ However, the underlying mechanism through which it is activated and its role in cervical cancer progression are still unclear. In this study, we demonstrated that, E6 activates lnc_000231 expression through promoting its promoter region H3K4me3 modification by destabilizing KDM5C. Functionally, we showed that lnc_000231 promotes cervical cancer cells proliferation and tumour formation by acting as miR‐497‐5p sponge and maintaining cyclin E1 (CCNE1) expression. Thus, this study identified lnc_000231 as a promising therapeutic target for cervical cancer.

## 2 MATERIAL AND METHODS

### 2.1 Cell lines

Cervical cancer cell lines HeLa, CaSki, SiHa, C‐33A and SW756 were purchased from American Type Culture Collection. Normal cervical epithelial cell line HcerEpic was purchased from ScienCell. HeLa, SiHa and C‐33A were cultured in Eagle's Minimum Essential Medium. CaSki was cultured in RPMI‐1640, and SW756 was cultured in Leibovitz's L‐15 Medium. HcerEpic was culture in cervical epithelial cell medium supplemented with cervical epithelial cell growth supplement.

### 2.2 RNA pull down

Biotin‐labelled lnc_000231 and miR‐497‐5p binding site mutated lnc_000231 synthesized from GenePharma were conjugated to streptavidin magnetic beads. Cells were lysed and pre‐incubated with streptavidin magnetic beads at 4℃ for 30 minutes. Streptavidin magnetic beads were then spun out. Supernatant was harvested and incubated with probe coated beads with shaking at 4℃ overnight. The next day, beads were extensively washed, and RNA was eluted with RNA elution buffer. The enrichment of miRNA was evaluated by qPCR.

### 2.3 Quantitative reverse transcription PCR

RNA was extracted by using miRNeasy Mini Kit (Cat No./ID: 217004; Qiagen, Hilden, Germany) according to manufacturer's instructions. cDNA was synthesized by using SuperScript™ IV Reverse Transcriptase (Cat: 18090010; Thermo Fisher, Waltham, USA). After reverse transcription, cDNAs were diluted 20 times and used as templates for real‐time PCR. Glyceraldehyde 3‐phosphate dehydrogenase (GAPDH) and U6 were used to normalize mRNA and miRNA expression, respectively. The relative RNA expression level was calculated with 2^−∆∆ct^.

### 2.4 Cell transfection

miR‐497‐5p mimic, siRNA targeting lnc_000231, scramble control and biotin‐labelled lnc_000231 were synthesized in GenePharma (Shanghai, China). Lipofectamine 3000 (Thermo Fisher, Waltham, USA) was used to transfect cells according to manufacturer's instructions. Functional studies were performed 48 hours after cell transfection.

### 2.5 Cell proliferation

After transfecting cells for 24 hours, cells were digested with trypsin and reseeded into six‐well plates at 0.1 millon/mL. Cell proliferation was monitored by counting cells at 2, 4, 6 and 8 days after cell growth.

### 2.6 Cell colony formation

Cell colony formation was used to detect cervical cancer cells ‘unlimited’ proliferation. Briefly, cells were harvested by trypsin digestion and serial diluted into 1000 cells/mL. ~500 cells were seeded into six‐well plates and allowed to grow for around 2 weeks until colonies can be clearly observed. One ml methanol was used to fix colonies at RT for 10 minutes. Colonies were then stained with 0.1% crystal violet at RT for another 5 minutes. After fixation, colonies were extensively washed with flowing water to minimize background staining, and colonies numbers were counted and statistically analysed.

### 2.7 Cell cycle

Cell cycle was detected with propidium iodide (PI) staining. In brief, cells were collected and washed with ice‐cold PBS twice. Cells were then resuspended with 100 µL PBS and fixed by dropping add 900 µL cold 100% ethanol into cell suspension with gentle vortex, followed by incubation at 4℃ for 2 hours. After fixation, cells were washed with PBS and stained with 1 mL PI staining solution (50 µg/mL; 1 mg/mL of RNase A, 0.1% Triton X‐100 in PBS) in the dark at room temperature for 30 minutes. Cell cycle was then detected by Fluorescence‐activated cell sorting (FACS).

### 2.8 Luciferase assay

Lnc_000231 or miR‐497‐5p promoter was amplified by PCR and cloned into pGL3 Vector. pGL3‐miR‐497‐5p or pGL3‐lnc_000231 was co‐transfected with Renilla vector and plasmids as indicated by using Lipofectamine 3000. Promoter activity was detected by Dual‐Luciferase reporter assay kit (Promega Corporation, Madison, USA). Renilla was used to normalize luciferase activity.

### 2.9 Western blotting

Cells were collected and washed with PBS twice. Cells were then lysed with RIPA lysis buffer containing protein inhibitors on ice for 30 minutes. Cell lysis was then spun down at 13 000 rpm for 30 minutes. Supernatant containing protein was collected, and protein was quantitated with BSA assay. SDS‐PAGE was used to separate proteins by electrophoresis based on protein size. After electrophoresis, protein was transferred to Polyvinylidene difluoride (PVDF) membrane. Membrane was blotted with 5% non‐fat milk at room temperature with shaking for 30 minutes. Primary antibodies targeting proteins of interest were incubated with membrane with shaking at 4℃ overnight. The next day, HRP‐tagged secondary antibodies were used to detect proteins of interest. Image was captured by Odyssey CLx.

### 2.10 CRISPR‐Cas9 mediated gene knockout

Cyclin E1 and KDM5C were deleted by CRISPR‐CAS9. Briefly, sgRNAs targeting CCNE1 and KDM5C were designed with CRISPRko from Broad Institute. sgRNA oligos were synthesized in GenePharma and cloned into pLentiGuide‐puro vector. Lentiviruses were packaged with pLentiGuide‐puro containing sgRNAs, VSVG and PsPAX2. Lentiviruses were then harvested 48 hours later and used to infect HeLa and SiHa cells stably expressing CAS9. After transduction, cells were selected with puromycin. Western blot was used to detect the efficiency of gene knockout at three days after puromycin selection.

### 2.11 Chromatin Immunoprecipitation quantitative PCR

Chromatin Immunoprecipitation quantitative PCR (ChIP‐qPCR) was performed as described previously.^27^ Briefly, 10 million cells were harvested and cross‐linked with 1% formaldehyde. DNA was fragmented by sonication. Protein‐DNA complexes were then pulled down by antibody of interest or IgG. Protein A/G beads were used to precipitate antibody‐protein‐DNA complexes, and DNA was step wisely washed with low salt wash buffer, high salt wash buffer, licl wash buffer and TE buffer. DNA was eluted with DNA elution buffer and reverse cross‐linked with NaCl. DNA was then purified, diluted and used for qPCR.

### 2.12 Northern blotting

Total RNA was extracted from cells by using miRNeasy Mini Kit (Cat No./ID: 217004; Qiagen) according to manufacturer's instructions. Extracted RNA was quantitated by spectrophotometry and resolved on 1% denaturing agarose gel. 32P‐labelled DNA probes targeting lnc_000231 was generated using a random oligonucleotide labelling kit (Amersham). Probe was incubated with membranes overnight at 65°C. The next day membrane was washed, and the radioactive signal was visualized by phosphorimaging and autoradiography.

### 2.13 Patient samples

Eighteen cervical cancer patients under surgery at Renmin Hospital of Wuhan University were involved in this study. Patients' samples were obtained during surgery and frozen down in liquid nitrogen for further study. This study was approved by the Ethics Committee of Renmin Hospital of Wuhan University. All the patients were informed consent in this study.

### 2.14 In vivo tumour formation

The animal experiment was strictly conducted according to the guidelines for the care and use of laboratory animals of china. Eighteen 4‐week‐old female BALB/c nude mice were purchased from Shanghai Animal Research Center. 1 × 10^7^ SiHa cells transfected with scramble or lnc_000231 siRNAs for 24 hours were resuspended in 500 µL PBS and injected into nude mice to establish xenograft models. When tumours reached about 30 mm^3^, tumour volumes were measured every week until the end of the experiment. The mice were killed on day 28, and tumour masses were excised and weighted.

### 2.15 Statistical analysis

SPSS was used for data analysis. Unpaired t test was applied to compare the differences between treated and untreated groups. All data were shown as mean ± SEM. *P < 0.05, **P < 0.01, ***P < 0.001. *P* < 0.05 was considered statistically significant.

## 3 RESULTS

### 3.1 Lnc_000231 is highly expressed in cervical cancer cells and patient tissues

**FIGURE 1:**
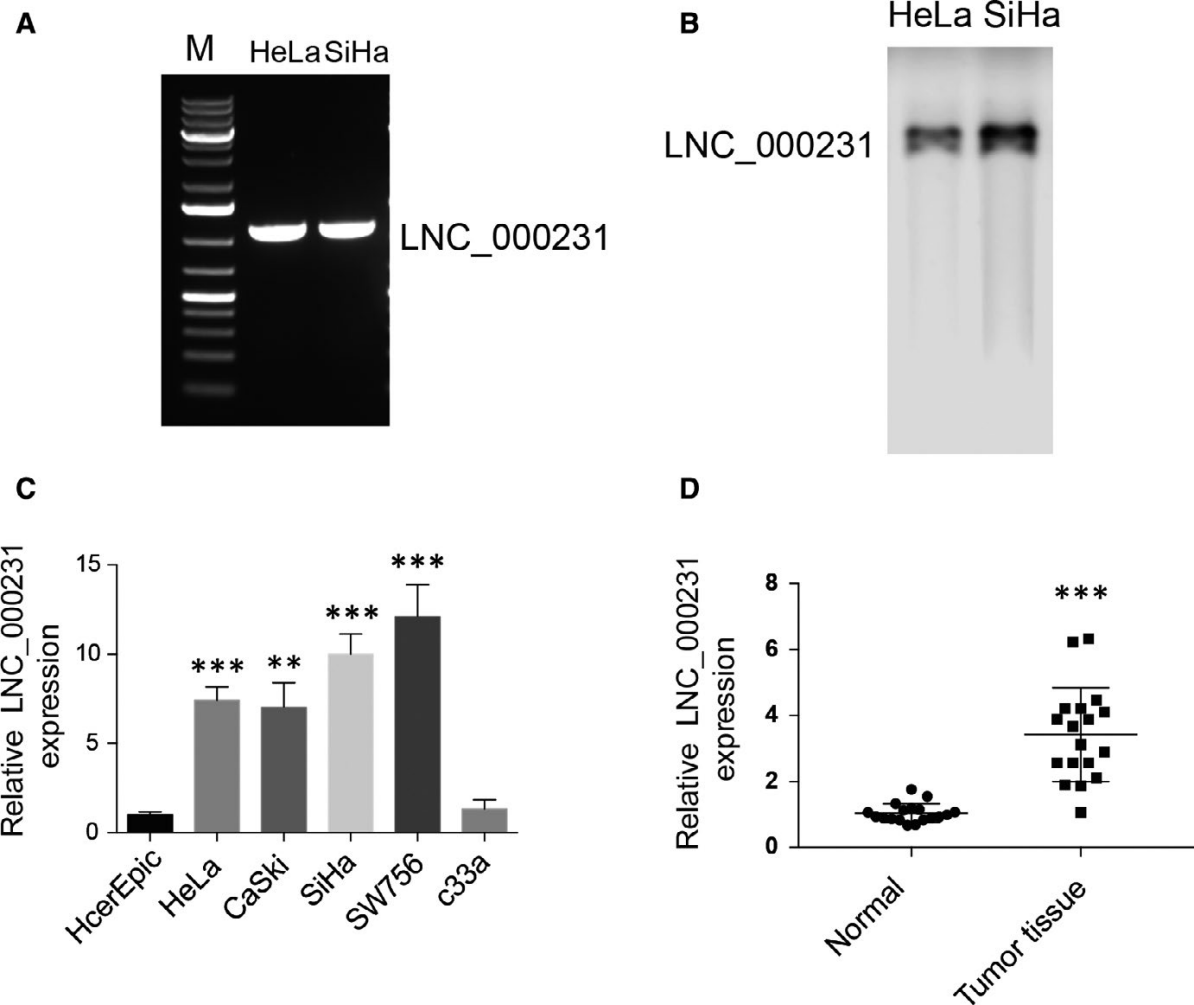
Detection of lnc_000231 expression. A, RNA was extracted, reversed into cDNA and was used to amplify lnc_000231 from HeLa and SiHa cells. B, Northern blotting detects lnc_000231 transcript from HeLa and SiHa cells. C, Quantitative reverse transcription PCR (RT‐qPCR) results of lnc_000231 in different cervical cancer cell lines. HcerEpic was normalized to 1. GAPDH was used as control. D, RT‐qPCR results of lnc_000231 in cervical cancer patient tumour tissues and adjacent normal tissues. GAPDH was used as control. **P < .01, ***P < .001.

High‐throughput RNA sequencing identified lnc_000231 was up‐regulated in cervical squamous cell carcinoma than its matched adjacent non‐tumour tissues.^26^ To confirm the expression of lnc_000231 in cervical cancer cells, RNA extracted from HeLa and SiHa cells was reverse transcribed into cDNA and used as template for PCR amplification. As shown in Figure 1A, lnc_000231 was readily detected in HeLa and SiHa cells. Northern blotting with probes against lnc_000231 confirmed this result (Figure 1B). The expression level of lnc_000231 in cervical cancer cells and patient tissues was then investigated. As indicated, lnc_000231 expression was significantly higher in most of the cervical cancer cell lines studied and tumour tissues than normal cervical epithelial cell line (Figure 1C, **P < 0.01, ***P < 0.001) and normal tissues (Figure 1D, ***P < 0.001), respectively. These results suggested that lnc_000231 is abundantly expressed in cervical cancer cells and tumour tissues.

### 3.2 E6 drives lnc_000231 expression in cervical cancer cells

**FIGURE 2:**
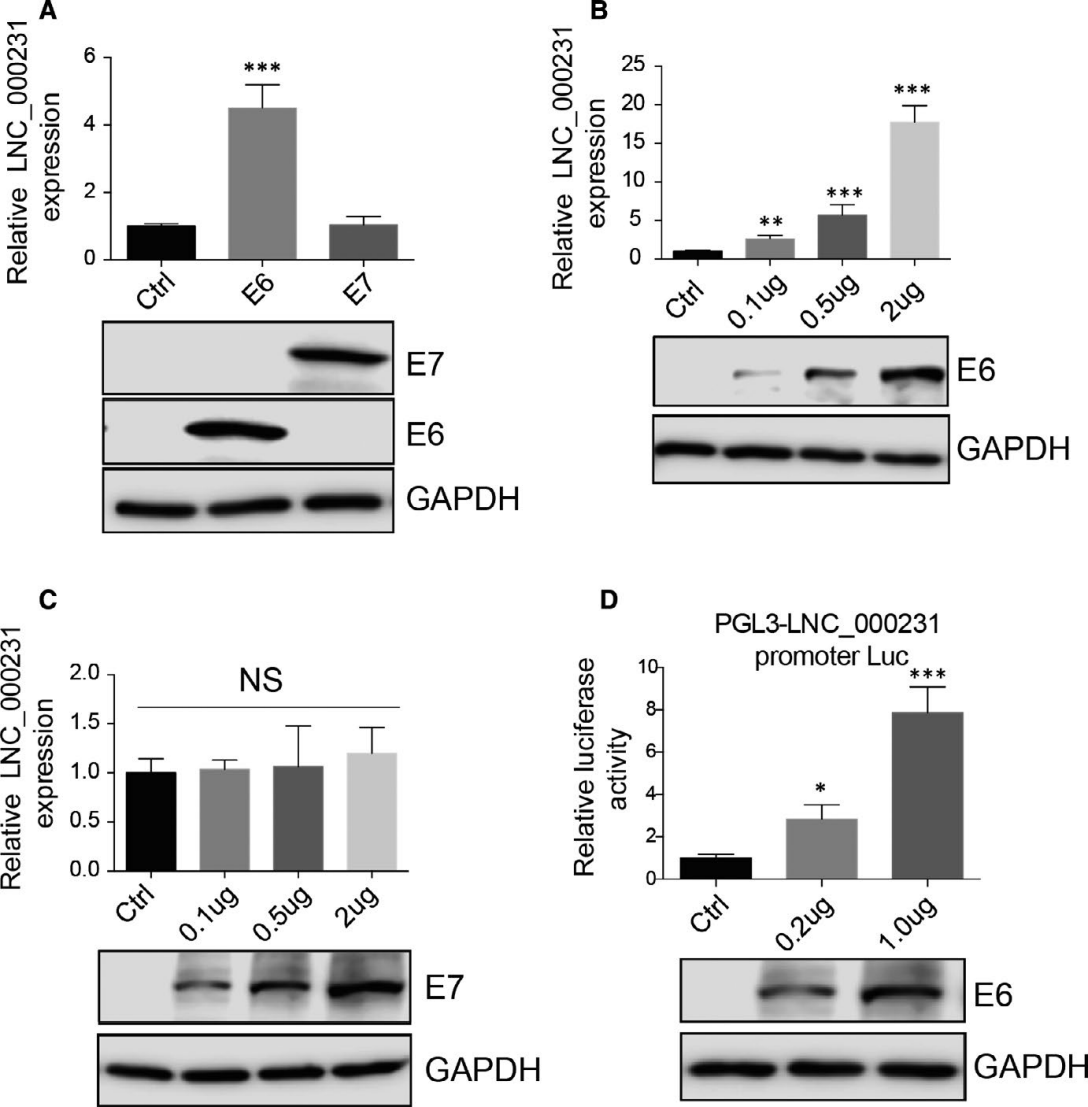
E6 up‐regulates lnc_000231 expression. A, Quantitative reverse transcription PCR (RT‐qPCR) detects lnc_000231 expression after exogenously overexpressing E6 or E7 in HcerEpic. The expression of E6 and E7 is indicated by Western blots, and GAPDH was used as control. B, RT‐qPCR detects lnc_000231 expression after exogenously overexpressing different amounts of E6 in HcerEpic. The expression of E6 is indicated by Western blots, and GAPDH was used as control. C, RT‐qPCR detects lnc_000231 expression after exogenously overexpressing different amounts of E7 in HcerEpic. The expression of E7 is indicated by Western blots, and GAPDH was used as control. D, Luciferase assay to determine lnc_000231 promoter activity in the presence of different doses of E6. *P < .05, **P < .01, ***P < .001

Human papillomavirus infection promotes cervical cancer progression. Next, we want to know whether increased lnc_000231 expression in cervical cancer is associated with HPV. Indeed, lnc_000231 expression is lower in HPV‐negative cervical cancer cell line c33a than other HPV‐positive cervical cancer cell lines (Figure 1C, **P < 0.01, ***P < 0.001), indicating HPV infection may promote lnc_000231 expression. E6 and E7 are HPV oncoproteins with transcriptional activity. Their role on lnc_000231 expression was first evaluated. cDNA expressing E6 or E7 was transfected into HcerEpic cells, and the expression of lnc_000231 was detected by quantitative reverse transcription PCR (RT‐qPCR). Exogenous expressing E6 but not E7 greatly up‐regulated lnc_000231 expression (Figure 2A, **P < 0.01), and E6 but not E7 promoted lnc_000231 expression in a dose‐dependent manner (Figure 2B,C, *P < 0.05, **P < 0.01). To further confirm this, lnc_000231 promoter was cloned into pGL3 vector and co‐transfected with different doses of E6. As indicated, E6 also increased lnc_000231 promoter activity in a dose‐dependent manner (Figure 2D, *P < 0.05, **P < 0.01). Thus, these results implicated that HPV oncoprotein E6 drives lnc_000231 expression in cervical cancer cells.

### 3.3 E6 promotes lnc_000231 promoter H3K27ac and H3K4me3 modification

**FIGURE 3:**
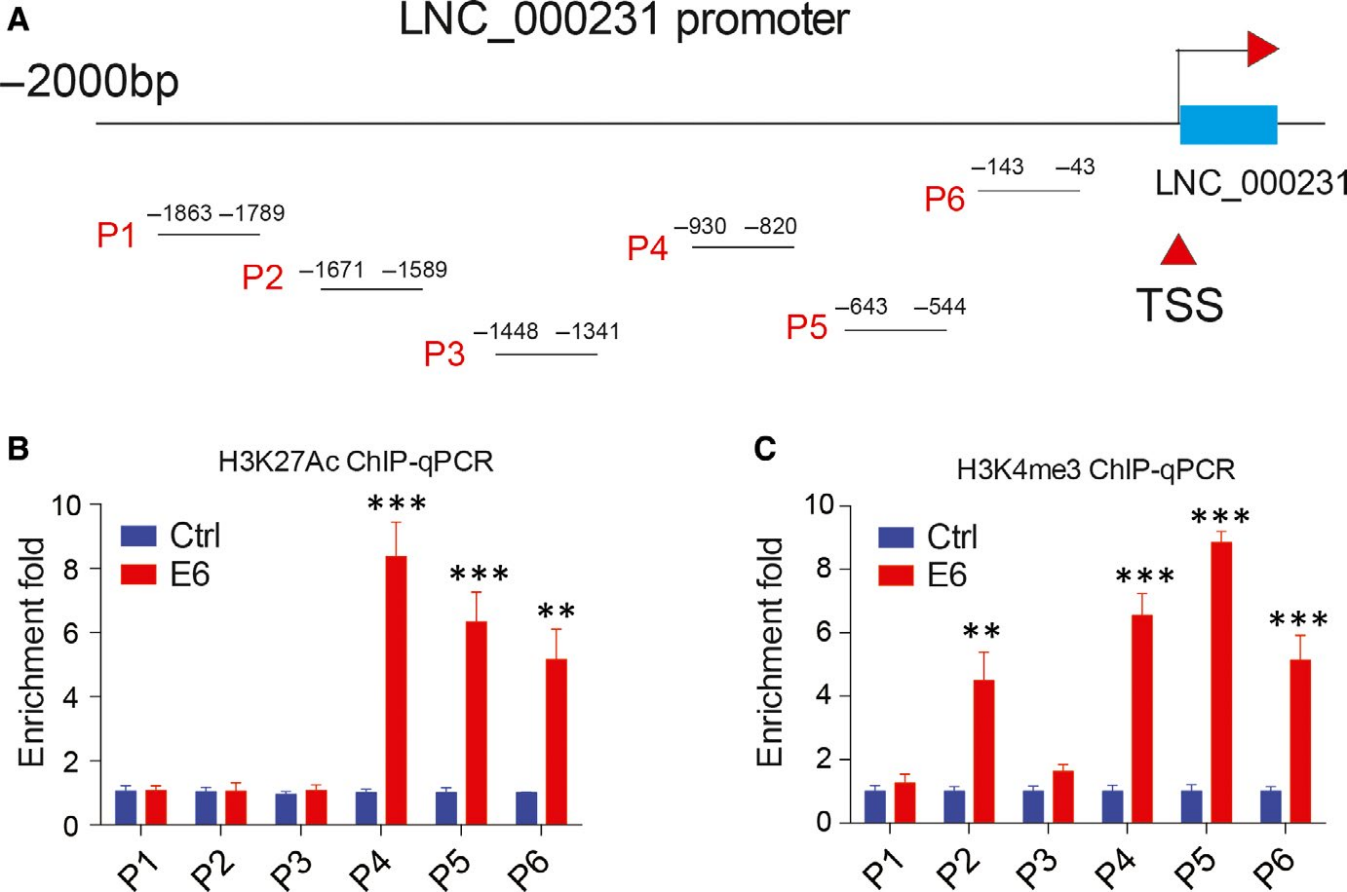
E6 promotes lnc_000231 promoter region activation marker H3K27ac and H3K4me3 modification. A, Diagram of lnc_000231 promoter. P1, P2, P3, P4, P5 and P6 are selected regions used for chromatin immunoprecipitation quantitative PCR (ChIP‐qPCR) amplification. Transcription start site (TSS) is indicated by red arrow. B, ChIP‐qPCR results of H3K27ac signal on different regions of lnc_000231 promoter in the presence or absence of E6. C, ChIP‐qPCR results of H3K4me3 signal on different regions of lnc_000231 promoter in the presence or absence of E6. **P < .01, ***P < .001

Histone H3K27ac and H3K4me3 modification indicate active gene transcription. The effect of E6 on lnc_000231 promoter H3K27ac and H3K4me3 modification was then examined. ChIP assay with antibodies against H3K27ac and H3K4me3 was performed in the presence or absence of E6. Primers spanning 2kb regions upstream of lnc_000231 TSS (Transcription start site) were used for qPCR. As indicated, overexpression of E6 significantly promoted lnc_000231 promoter region, especially regions close to lnc_000231 TSS H3K27ac and H3K4me3 modification (Figure 3B,C, *P < 0.05, **P < 0.01).

### 3.4 E6 promotes lnc_000231 promoter H3K4me3 modification through destabilizing KDM5C

**FIGURE 4:**
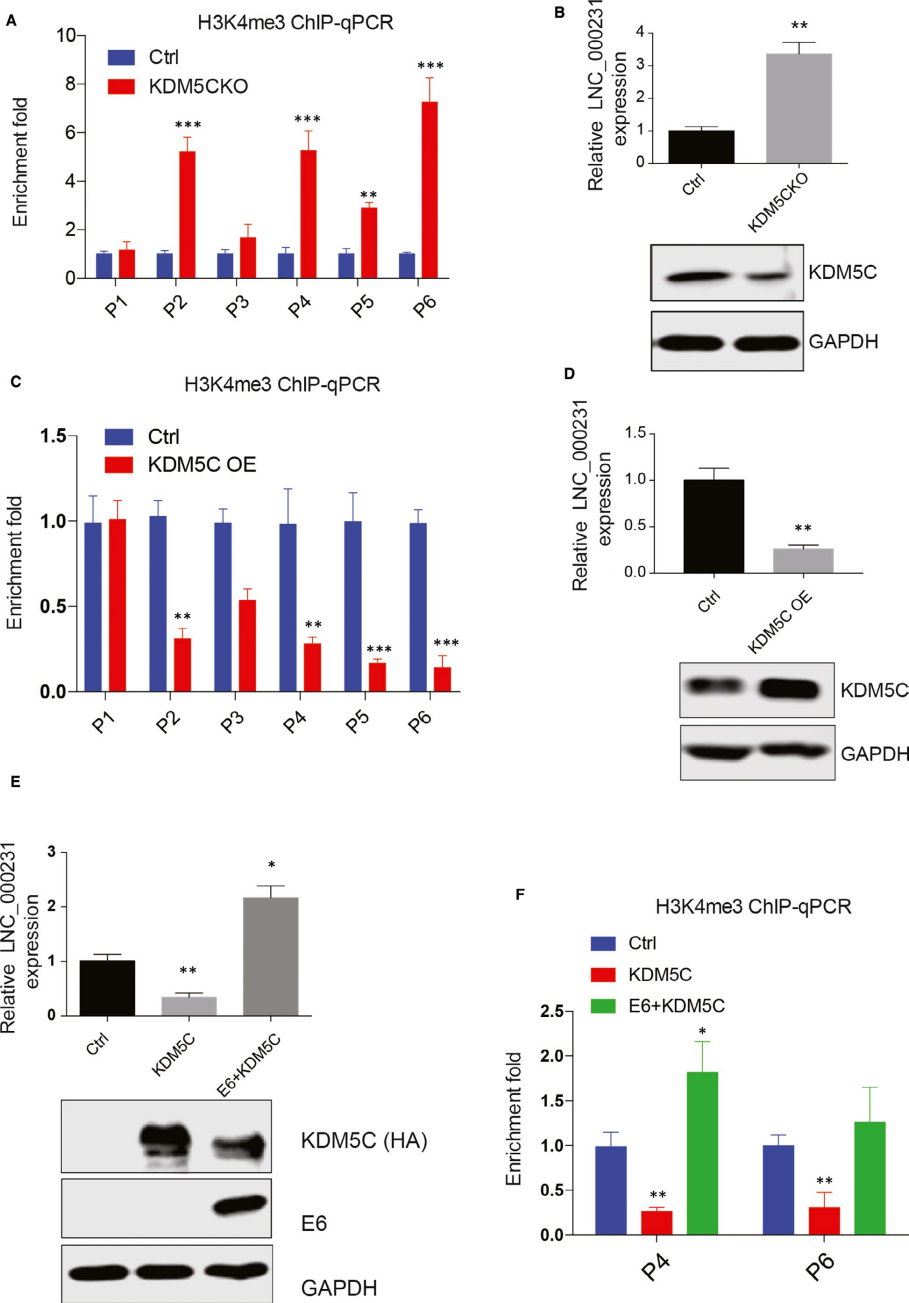
E6 promotes lnc_000231 promoter H3K4me3 modification by destabilizing KDM5C. A, Chromatin immunoprecipitation quantitative PCR (ChIP‐qPCR) results of H3K4me3 signal on lnc_000231 promoter after knocking down KDM5C in HeLa cells. B, Lnc_000231 expression in HeLa cells after depletion of KDM5C. The decreased expression of KDM5C was indicated by Western blots. C, ChIP‐qPCR results of H3K4me3 signal on lnc_000231 promoter after overexpressing KDM5C in HeLa cells. D, Lnc_000231 expression in HeLa cells after overexpressing KDM5C. The expression of KDM5C was indicated by Western blots. E, Lnc_000231 expression after transfecting KDM5C or co‐transfecting KDM5C and E6 into HcerEpic. The expression of E6 and HA tagged KDM5C is indicated by Western blots. F, Lnc_000231 promoter H3K4me3 modification after transfecting KDM5C or co‐transfecting KDM5C and E6 into HcerEpic. Lnc_000231 promoter P4 and P6 region were selected for study. *P < .05, **P < .01, ***P < .001

KDM5C is a histone demethylase that removes H3K4 methylation, thereby playing a central role in H3K4 methylation.^28^ Previous studies showed that E6 interacts with KDM5C in cervical cancer cells and promotes KDM5C degradation in an E3 ligase E6AP‐ and proteasome‐dependent manner.^29^ This leads us to ask whether E6 promotes lnc_000231 promoter H3K4me3 modification is mediated through destabilizing KDM5C. sgRNA targeting KDM5C was used to delete KDM5C in HeLa cells. The effect of KDM5C knockout on lnc_000231 promoter H3K4me3 modification was evaluated by ChIP‐qPCR. Interestingly, KDM5C knockout greatly up‐regulated lnc_000231 promoter H3K4me3 modification (Figure 4A, *P < 0.05, **P < 0.01), and the expression of lnc_000231 was also increased (Figure 4B, **P < 0.01). In contrast, overexpressing KDM5C significantly down‐regulated lnc_000231 promoter H3K4me3 modification (Figure 4C, *P < 0.05, **P < 0.01) and lnc_000231 expression (Figure 4D, *P < 0.05, **P < 0.01). These results suggested that KDM5C is responsible for lnc_000231 promoter H3K4me3 modification. KDM5C tagged with HA was then co‐transfected with or without E6, and their effects on lnc_000231 expression and lnc_000231 promoter H3K4me3 modification were investigated. As expected, overexpressing KDM5C down‐regulated lnc_000231 expression. However, lnc_000231 expression was completely restored in the presence of E6 (Figure 4E, *P < 0.05, **P < 0.01). In addition, the decreased H3K4me3 signal on lnc_000231 promoter induced by KDM5C was also rescued by E6 (Figure 4F, *P < 0.05, **P < 0.01). These results indicated that E6 promotes lnc_000231 promoter H3K4me3 modification and lnc_000231 expression at least partially through destabilizing KDM5C.

### 3.5 lnc_000231 promotes cervical cancer cell proliferation, colony formation, cell cycle progression and in vivo tumour formation

**FIGURE 5:**
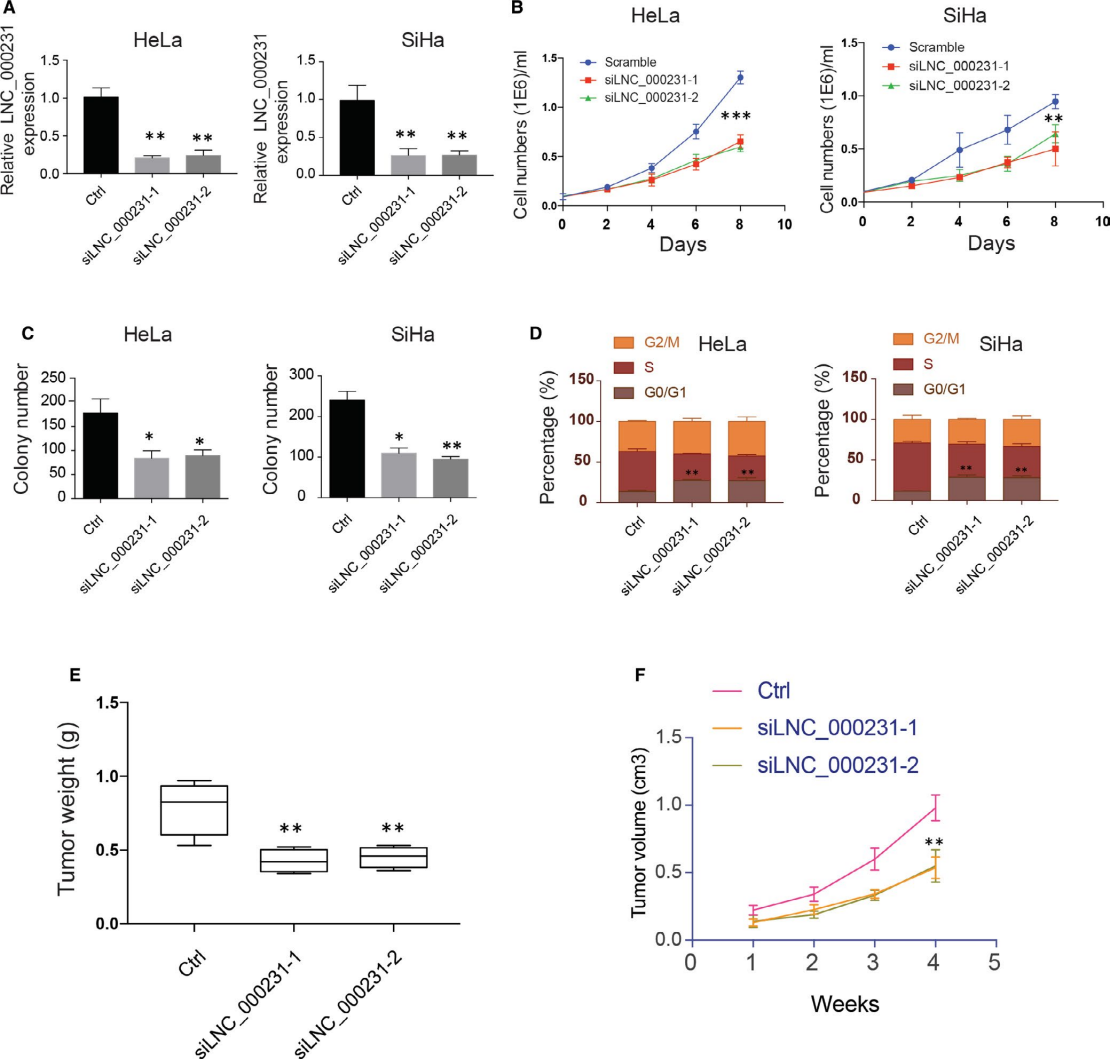
lnc_000231 promotes cervical cancer cell proliferation, colony formation, cell cycle progression and in vivo tumour formation. A, Lnc_000231 expression in HeLa and SiHa cells after silencing lnc_000231 by siRNAs. B, Hela and SiHa cell growth after silencing lnc_000231. C, Detection of Hela and SiHa cell colony formation after silencing lnc_000231. D, Evaluation of Hela and SiHa cell cycle after silencing lnc_000231. E, Tumour weights and (F) tumour growth curve after injecting lnc_000231 knockout or wild‐type SiHa cells into nude mice (n = 6). *P < .05, **P < .01, ***P < .001

To determine whether lnc_000231 plays a role in cervical cancer progression, siRNAs targeting lnc_000231 were used to knock‐down lnc_000231. The effect of lnc_000231 knockdown on HeLa and SiHa cells proliferation, cell colony formation and cell cycle progression was first evaluated. As indicated, siRNAs efficiently down‐regulated lnc_000231 expression (Figure 5A, *P < 0.05, **P < 0.01). In addition, cell growth, cell colony formation and cell cycle progression were also impaired (Figure 5B‐D, *P < 0.05, **P < 0.01). To further understand whether lnc_000231 also plays a role in vivo. SiHa cells with or without lnc_000231 knockdown were injected into nude mice, tumour formation was then monitored. In line with in vitro results, knockdown of lnc_000231 greatly impaired SiHa cell tumour formation in vivo (Figure 5E,F, *P < 0.05, **P < 0.01). Combined with the in vivo and in vitro data, we can infer that lnc_000231 plays an important role in the development of cervical cancer formation.

### 3.6 lnc_000231 down‐regulates miR‐497‐5p expression in vitro and in vivo

**FIGURE 6:**
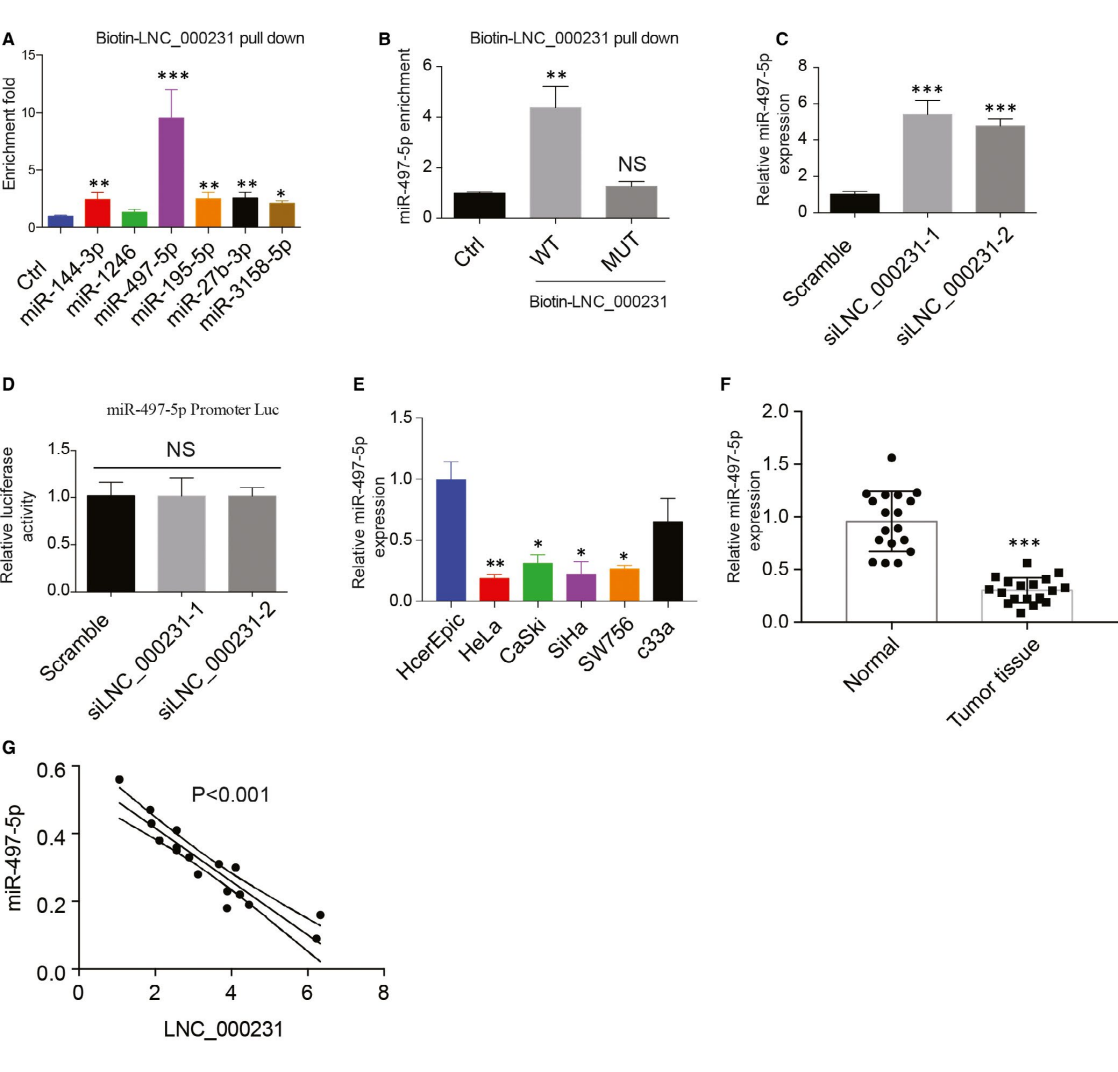
lnc_000231 down‐regulates miR‐497‐5p expression in vitro and in vivo. A, Quantitative reverse transcription PCR (RT‐qPCR) detects indicated miRNA expression after pulling down by biotin‐labelled lnc_000231. B, Pull down miR‐497‐5p with biotin‐labelled wild‐type lnc_000231 or miR‐497‐5p binding sites mutated lnc_000231 from Hela cell lysates. C, Detection of miR‐497‐5p expression after silencing lnc_000231. D, Luciferase assay detects miR‐497‐5p promoter activity after silencing lnc_000231. E, miR‐497‐5p expression in cervical cancer cell lines and HcerEpic. F, miR‐497‐5p expression in cervical cancer patient tumour tissues and adjacent normal tissues. G, Correlation analysis of miR‐497‐5p and lnc_000231 in cervical cancer patients. *P < .05, **P < .01, ***P < .001

Increasing evidence suggests that lncRNA regulates miRNA expression and affects miRNA targeted gene function.^30^ We hypothesis that lnc_000231 regulates miRNA expression in cervical cancer cells. Previous genome‐wide RNA sequencing and analysis showed that lnc_000231 may interact with miR‐144‐3‐p, miR‐1246, miR‐497‐5p, miR‐195‐5p, miR‐27b‐3p and mR‐3158‐5p in cervical cancer. To validate this, biotin‐labelled lnc_000231 was used to precipitate miRNAs from cervical cancer cell lysates. The expression level of each miRNA was examined by qPCR. Interestingly, among all the miRNA tested, miR‐497‐5p showed the highest binding affinity with lnc_00023 (Figure 6A, *P < 0.05, **P < 0.01), indicating miR‐497‐5p may be the target of lnc_00023 in cervical cancer cells. To confirm this, site‐directed mutation was used to generate lnc_00023 mutants in which miR‐497‐5p binding site was mutated. lnc_00023 WT and mutants were then used to pull down miR‐497‐5p. As expected, WT lnc_00023 efficiently precipitated miR‐497‐5p; however, lnc_00023 mutants failed to pull down miR‐497‐5p (Figure 6B, **P < 0.01), confirming lnc_00023 interacts with miR‐497‐5p in vivo. siRNA was used to knock‐down lnc_00023, and the expression of miR‐497‐5p was evaluated. Interestingly, silencing lnc_00023 significantly increased miR‐497‐5p expression (Figure 6C, *P < 0.05, **P < 0.01). To eliminate the possibility that lnc_00023 down‐regulates miR‐497‐5p expression through inhibiting its transcription, miR‐497‐5p promoter region was cloned into PGL3 vector and co‐transfected with or without siRNAs targeting lnc_00023. As indicated, silencing lnc_00023 did not affect miR‐497‐5p promoter activity (Figure 6D), suggesting lnc_00023 regulates miR‐497‐5p expression through post‐transcription. The expression of miR‐497‐5p in cervical cancer cells and tumour tissues was also studied. In contrast to lnc_000231, miR‐497‐5p expression was significantly lower in cervical cancer cells and tumour tissues compared with normal cervical epithelial cell line (Figure 6E, **P < 0.01, ***P < 0.001) and normal tissues (Figure 6F, ***P < 0.001), respectively. In addition, correlation analysis showed that, miR‐497‐5p expression was negatively correlated with lnc_00023 in patient tumour tissues (Figure 6G, ***P < 0.001). In summary, these results clearly demonstrated that lnc_000231 down‐regulates miR‐497‐5p expression in cervical cancer cells (in vitro) and patient tumour tissues (in vivo).

### 3.7 lnc_000231 rescues CCNE1 expression and cervical cancer cell growth by suppressing miR‐497‐5p

**FIGURE 7:**
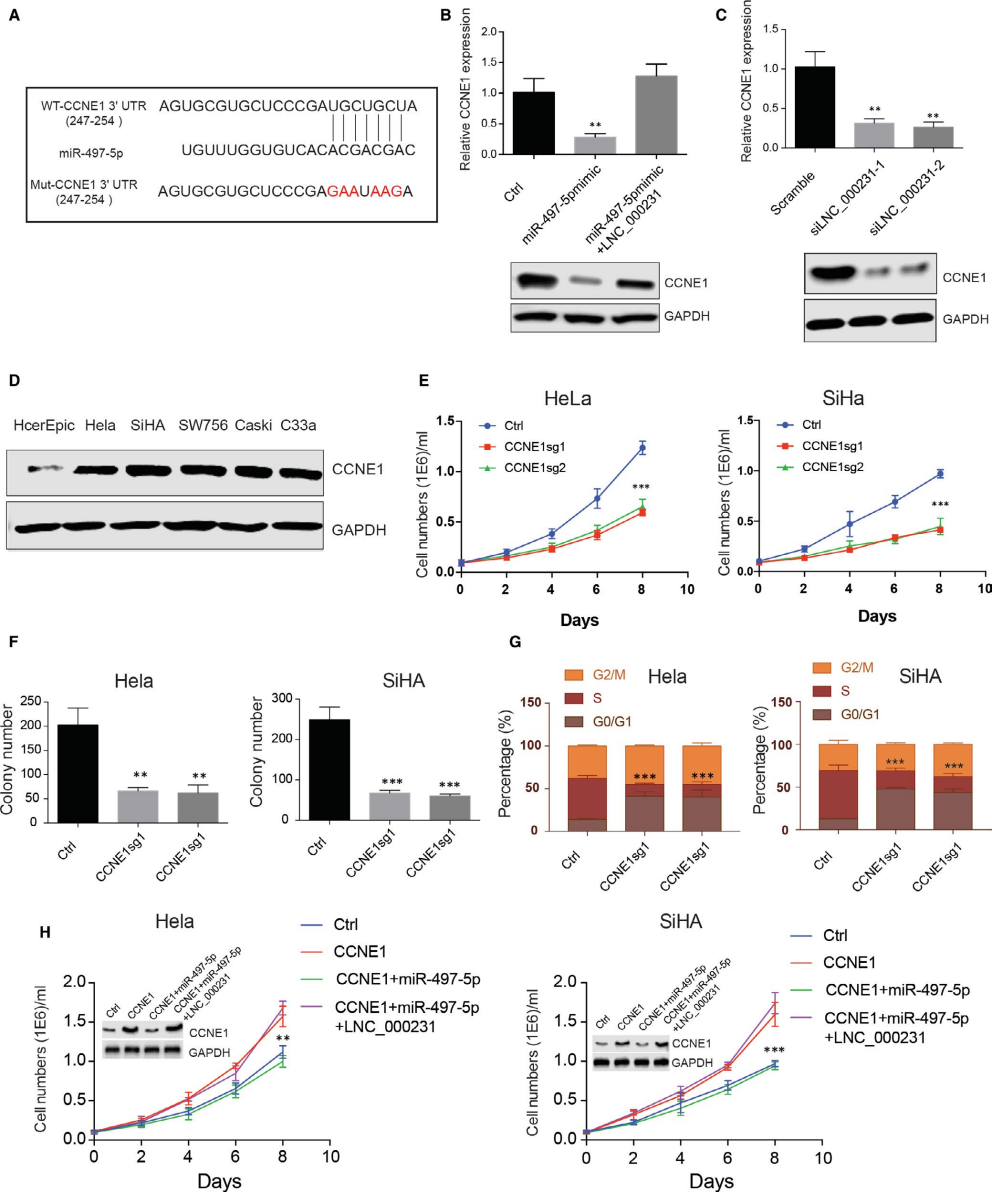
lnc_000231 rescues cyclin E1 (CCNE1) expression and cervical cancer cell growth by suppressing miR‐497‐5p. A, Diagram of CCNE1 3ʹUTR and miR‐497‐5p interactions. B, CCNE1 expression after transfecting miR‐497‐5p mimic or co‐transfecting miR‐497‐5p mimic and lnc_000231 into HeLa cells. C, CCNE1 expression after silencing lnc_000231. D, Detection of CCNE1 expression across different cervical cancer cell lines. Detection of HeLa and SiHa cell growth (E), cell colony formation (F) and cell cycle progression (G) after knocking down CCNE1. H, Evaluation of cell growth after transfecting CCNE1, CCNE1 and miR‐497‐5p or CCNE1, miR‐497‐5p and lnc_000231 in HeLa and SiHa cells. The expression of CCNE1 was indicated by Western blots. **P < .01, ***P < .001

MicroRNA regulates gene expression through binding to its target genes three prime untranslated region (3'‐UTR) and repress protein production by destabilizing mRNA. Next, we seek to understand which gene is targeted by miR‐497‐5p in cervical cancer cells. TargetScan was then applied to predict miR‐497‐5p targets. Cyclin E1, a cyclin gene essential for the control of the cell cycle at the G1/S transition, was shown to be targeted by miR‐497‐5p. The binding sites of miR‐497‐5p to CCNE1 3'‐UTR was shown (Figure 7A, **P < 0.01). miR‐497‐5p mimic was used to study the regulatory roles of miR‐497‐5p on CCNE1. Exogenously overexpressing miR‐497‐5p greatly decreased CCNE1 expression in both protein and transcription level. However, co‐transfecting miR‐497‐5p and lnc_000231 restored CCNE1 expression (Figure 7B, **P < 0.01). In addition, when silencing lnc_000231 expression in HeLa cells, CCNE1 expression was also impaired (Figure 7C, **P < 0.01). These results suggested that lnc_000231 may up‐regulate CCNE1 expression through inhibiting miR‐497‐5p. As a cell cycle regulator, the roles of CCNE1 in cervical cancer were also investigated. Western blot was first used to detect CCNE1 expression. Results showed that CCNE1 was highly expressed in cervical cancer cells than normal cervical epithelial cell line (Figure 7D). Further knocking down CCNE1 by using sgRNAs greatly decreased HeLa and SiHa cell growth, cell colony formation and cell cycle progression from G1 to S phase (Figure 7E‐G, **P < 0.01, ***P < 0.001). Moreover, overexpressing CCNE1 greatly promoted HeLa and SiHa cell growth. In addition to that, co‐transfecting CCNE1 and miR‐497‐5p suppressed CCNE1‐induced cell growth; however, co‐transfecting CCNE1, miR‐497‐5p and lnc_000231 restored cell growth (Figure 7H, **P < 0.01, ***P < 0.001). Thus, these results suggested that in the cervical cancer cells, lnc_000231 function as a sponge of miR‐497‐5p to maintain CCNE1 expression.

## 4 DISCUSSION

Emerging evidence suggest that lncRNA play important roles in disease development, particular in cancers.^22^ By serving as potential cancer diagnostic markers or therapeutic targets, lncRNA has caused great attention in recent years. Previous genome‐wide sequencing showed that lnc_000231 was up‐regulated in cervical cancer tumour tissues than adjacent normal tissues.^26^ However, the biological significance of lnc_000231 in cervical cancer is still unknown. In this study, we demonstrated that lnc_000231 activation in cervical cancer cells is mediated through E6. E6 degrades KDM5C promoted lnc_000231 promoter region H3K4me3 modification and lnc_000231 expression. In the meantime, we showed that lnc_000231 plays a critical role in cervical cancer progression by acting as miR‐497‐5p sponge and maintain CCNE1 expression. These results provided new insights into the molecular basis of cervical cancer.

E6 and E7 are the most studied oncoproteins during HPV infection. E6 regulates gene expression through multiple layers. Through paring with ubiquitin ligase E6AP, E6 promotes many proteins degradation including TP53, TIP60, hADA3, MGMT, BAK and BRCA1.^17^, ^31^, ^32^, ^33^ By acting as a transcription factor, E6 can also up‐regulate some oncogenes like epidermal growth factor receptor (EGFR)^34^ and c‐MET.^35^ Recent studies showed that E6 activates EGFR and c‐MET super‐enhancers by destabilizing KDM5C. In this study, we first detected lnc_000231 expression across different cervical cancer cell lines, and we found that lnc_000231 was highly expressed in most of the cervical cancer cells but not in c33a, an HPV‐negative cervical cancer cell line. This leads us to infer that HPV infection may be the cause of lnc_000231 activation. Indeed, when we exogenously expressing E6 in normal cervical epithelial cell line, lnc_000231 expression was also up‐regulated, confirming that E6 drives lnc_000231 expression in cervical cancer cells.

Histone H3K27ac and H3K4me3 modification indicate active transcription. Here, we showed that exogenously overexpressing E6 greatly promoted lnc_000231 promoter H3K27ac and H3K4me3 modification and promoter activation, indicating E6 regulates lnc_000231 at transcription level. As a histone demethylase which removes H3K4 methylation, the roles of KDM5C in lnc_000231 promoter H3K4 modification were also investigated. Interestingly, we found that overexpressing KDM5C greatly suppressed lnc_000231 promoter H3K4me3 modification and lnc_000231 expression. However, co‐expressing E6 and KDM5C restored H3K4me3 modification and lnc_000231 expression, suggesting E6 promotes lnc_000231 promoter H3K4me3 modification through destabilizing KDM5C.

In order to explore the roles of lnc_000231 in cervical cancers, we examined the effect of lnc_000231 knockout on cervical cancer cells and in vivo tumour formation. siRNAs targeting lnc_000231 were used to knockout lnc_000231. siRNA efficiently knocked down lnc_000231 in HeLa and SiHa cells. Cell growth, cell colony formation, cell cycle progression and in vivo tumour formation were also greatly impaired after knocking down lnc_000231, suggesting lnc_000231 is a potential target for cervical cancer treatment.

Long non‐coding RNA has been proposed to function in multiple directions, such as nuclear domain organization, transcriptional regulation and post‐transcriptional modification.^36^, ^37^ Recently, more and more studies have shown that lncRNA function as miRNA sequester and affect miRNA target gene expression. For example, lncRNA XLOC_006390 has been reported to facilitate cervical cancer tumorigenesis by function as a ceRNA against miR‐331‐3p and miR‐338‐3p.^38^ Long non‐coding RNA‐TCONS_00026907 inhibits miR‐143‐5p expression and promotes the progression and prognosis of cervical cancer.^39^ In concert with these studies, here, we showed that lnc_000231 could directly interact with miR‐497‐5p in cervical cancer cells. Contrary to lnc_000231, miR‐497‐5p expression was down‐regulated in cervical cancer cells and tumour tissues and was negatively correlated with lnc_000231 in cervical cancer tumour tissues. In addition, knocking down lnc_000231 significantly up‐regulated miR‐497‐5p expression, suggesting lnc_000231 suppress miR‐497‐5p expression in cervical cancer.

Cyclin E1 is a member of the highly conserved cyclin family. Within the cell, CCNE1 forms a complex with and regulates the activity of CDK2, which further promotes cell cycle G1/S transition. Amplification of CCNE1 is associated with many cancer progressions including gastric cancer, bladder cancer, ovarian cancer and triple negative breast cancer and linked to poor prognosis.^40^, ^41^, ^42^, ^43^ In this study, we found that CCNE1 is up‐regulated in cervical cancer cells. Functional studies showed that CCNE1 play critical roles in cervical cancer cell growth and cell cycle transition from G1to S phase. Bioinformatic analysis and experimental validation showed that CCNE1 is a target of miR‐497‐5p. Overexpressing miR‐497‐5p greatly down‐regulated CCNE1 expression in cervical cancer cells. However, the impaired CCNE1 expression and cell growth induced by miR‐497‐5p was completely restored in the presence of lnc_000231.

In summary, in this study we found a new signalling pathway through which E6 promotes cervical cancer progression. E6 hijacked KDM5C/lnc_000231/miR‐497‐5p/CCNE1 signalling pathway is promising targets for cervical cancer treatment in the future.

## CONFLICT OF INTERESTS

The authors declare that they have no competing interests.

## AUTHOR CONTRIBUTION

**Yan Zhang:** Conceptualization (equal); Project administration (equal); Supervision (equal). **Xing Li:** Data curation (equal); Investigation (equal); Software (equal). **Jun Zhang:** Methodology (equal); Validation (equal). **Lin Mao:** Formal analysis (equal); Validation (equal); Visualization (equal).

## Data Availability

The authors confirm that the data supporting the findings of this study are available within the article.
